# Functionally selective activation of the dopamine receptor D_2_ is mirrored by the protein expression profiles

**DOI:** 10.1038/s41598-021-83038-x

**Published:** 2021-02-10

**Authors:** Deborah Wenk, Vladimir Ignatchenko, Andrew Macklin, Harald Hübner, Peter Gmeiner, Dorothée Weikert, Monika Pischetsrieder, Thomas Kislinger

**Affiliations:** 1 Food Chemistry, Department of Chemistry and Pharmacy, Friedrich-Alexander-Universität Erlangen-Nürnberg (FAU), Nikolaus-Fiebiger-Str. 10, 91058 Erlangen, Germany; 2 Princess Margaret Cancer Centre, University Health Network, 101 College Street, Toronto, ON Canada; 3 Medicinal Chemistry, Department of Chemistry and Pharmacy, Friedrich-Alexander-Universität Erlangen-Nürnberg (FAU), Nikolaus-Fiebiger-Str. 10, 91058 Erlangen, Germany; 4 Department of Medical Biophysics, University of Toronto, 101 College Street, Toronto, ON Canada

**Keywords:** Biological techniques, Cell biology, Drug discovery

## Abstract

The development of functionally selective or biased ligands is a promising approach towards drugs with less side effects. Biased ligands for G protein-coupled receptors can selectively induce G protein activation or β-arrestin recruitment. The consequences of this selective action on cellular functions, however, are not fully understood. Here, we investigated the impact of five biased and balanced dopamine D_2_ receptor agonists and antagonists on the global protein expression in HEK293T cells by untargeted nanoscale liquid chromatography–tandem mass spectrometry. The proteome analysis detected 5290 protein groups. Hierarchical clustering and principal component analysis based on the expression levels of 1462 differential proteins led to a separation of antagonists and balanced agonist from the control treatment, while the biased ligands demonstrated larger similarities to the control. Functional analysis of affected proteins revealed that the antagonists haloperidol and sulpiride regulated exocytosis and peroxisome function. The balanced agonist quinpirole, but not the functionally selective agonists induced a downregulation of proteins involved in synaptic signaling. The β-arrestin-preferring agonist BM138, however, regulated several proteins related to neuron function and the dopamine receptor-mediated signaling pathway itself. The G protein-selective partial agonist MS308 influenced rather broad functional terms such as DNA processing and mitochondrial translation.

## Introduction

G protein-coupled receptors (GPCRs) are the largest class of receptors in the human genome^1^, with more than 800 members expressed in every organ system^2^. Initially, GPCRs were assumed to signal predominantly through G proteins, with β-arrestins being responsible for GPCR desensitization^3^. It was demonstrated, however, that β-arrestins can also activate signaling cascades independent of G protein activation through multiple mediators such as mitogen-activated protein kinases (MAPKs) or phosphoinositide 3-kinase (PI3K)^4^. Ligands that equally activate all available signaling pathways controlled by a GPCR are termed “balanced ligands”, whereas biased ligands preferentially activate a subset of the signal transducers, e.g. G protein signaling or β-arrestin-mediated pathways^5^. Since G protein- and β-arrestin-mediated signaling have distinct biochemical and physiological effects^1^, the development of biased ligands may lead to drugs with a more specific activity profile and reduced side effects^3^. This hypothesis has been supported by the therapeutic benefit of several functionally selective GPCR ligands, for example in the treatment of schizophrenia^6^, pain^7^ or congestive heart failure^8^.

Several assays have been developed to examine different aspects of ligand-induced receptor activation, such as the GPCR’s proximity to a primary signal transducer like β-arrestin or receptor redistribution (e.g. internalization)^1^. Most assays that evaluate GPCR signaling focus on single steps of cellular signal transduction, like G protein activation, the accumulation of second messengers or the phosphorylation of signal mediators such as MAPKs or AKT^1^. Although these methods enable the precise characterization of receptor-activation profiles, results gained at different steps of the signaling cascade may be difficult to compare because the measured signals are amplified differently^1^. Methods like dynamic mass redistribution and cellular impedance assays, on the other hand, measure the cell’s integrated response to GPCR activation allowing for a fast assessment whether the ligand activates a given GPCR along any available signaling pathway. A suitable approach for a comprehensive evaluation of the cellular effects of receptor–ligand interaction is the analysis of cellular protein expression upon the binding of ligands to GPCRs. Proteome changes reflect the overall cellular consequences of specific signaling pathways and the functional analysis of affected proteins can be used to uncover biological processes regulated by ligands.

So far, few studies employed protein expression analysis to investigate cellular effects induced by GPCR signaling. Instead, proteomics techniques were used, for example, to analyze ligand-induced GPCR phosphorylation patterns^9,10^. Furthermore, direct interaction partners of GPCRs were identified by immunoprecipitation^11^ or pull-down strategies^12^. Another proteomics method analyzes rapid phospho-proteome changes in response to receptor stimulation to reveal the induced signaling pathways. This method elucidated, for example, brain signaling prompted by the activation of the dopamine receptor D_1_^13^ or the kappa opioid receptor^14^ in vivo. In contrast, the analysis of cellular protein expression at later stages focuses on the effects induced by GPCR signaling. Thus, Emirbayer et al. revealed an enrichment of cellular effects such as cytokine–cytokine receptor interactions and acute inflammatory processes in histamine-treated endothelial cells after 24 h^15^. Dong et al. used proteome analysis to investigate cellular effects induced by the stimulation of the orphan GPCR bombesin-like receptor 3 for one to twelve hours, indicating a regulation of cell death and protein synthesis, particularly mRNA translation^16^.

The present proteome profiling investigated cellular consequences induced by differential stimulation of the dopamine D_2_ receptor (D_2_R). D_2_R is a GPCR of clinical relevance, which is involved, amongst others, in the pathogenesis of schizophrenia^17^ and Parkinson’s disease^18^. Its ligands are used for the treatment of these and other neurological disorders like restless legs syndrome^19^. In addition to clinically applied D_2_R antagonists and balanced agonists, functionally selective D_2_R agonists have been developed that preferentially induce G protein activation or β-arrestin recruitment^6,20–22^. While antagonism for D_2_R-mediated recruitment of β-arrestin has been described as a common property of clinically effective antipsychotics^23^, selective targeting of this signaling pathway has been proposed as a novel strategy for alleviating l-3,4-dihydroxyphenylalanine (l-DOPA)-induced dyskinesia in the treatment of Parkinson’s disease^24^. However, β-arrestin-biased D_2_R ligands were also reported to act as effective antipsychotics^6^.

To elucidate the cellular impact of balanced and biased D_2_R activation, we stimulated an in vitro cell model expressing the short isoform of D_2_R (D_2S_R) with balanced or functionally selective D_2_R ligands and determined the quantitative changes of intracellular proteins by label-free proteome analysis. The subsequent bioinformatics analysis of the differential protein expression including functional enrichment analysis investigated the specific cellular effects induced by each ligand type. We assessed the extent to which the functional selectivity determined by assays evaluating primary signal transduction is mirrored at the proteome level.

**Figure 1:**
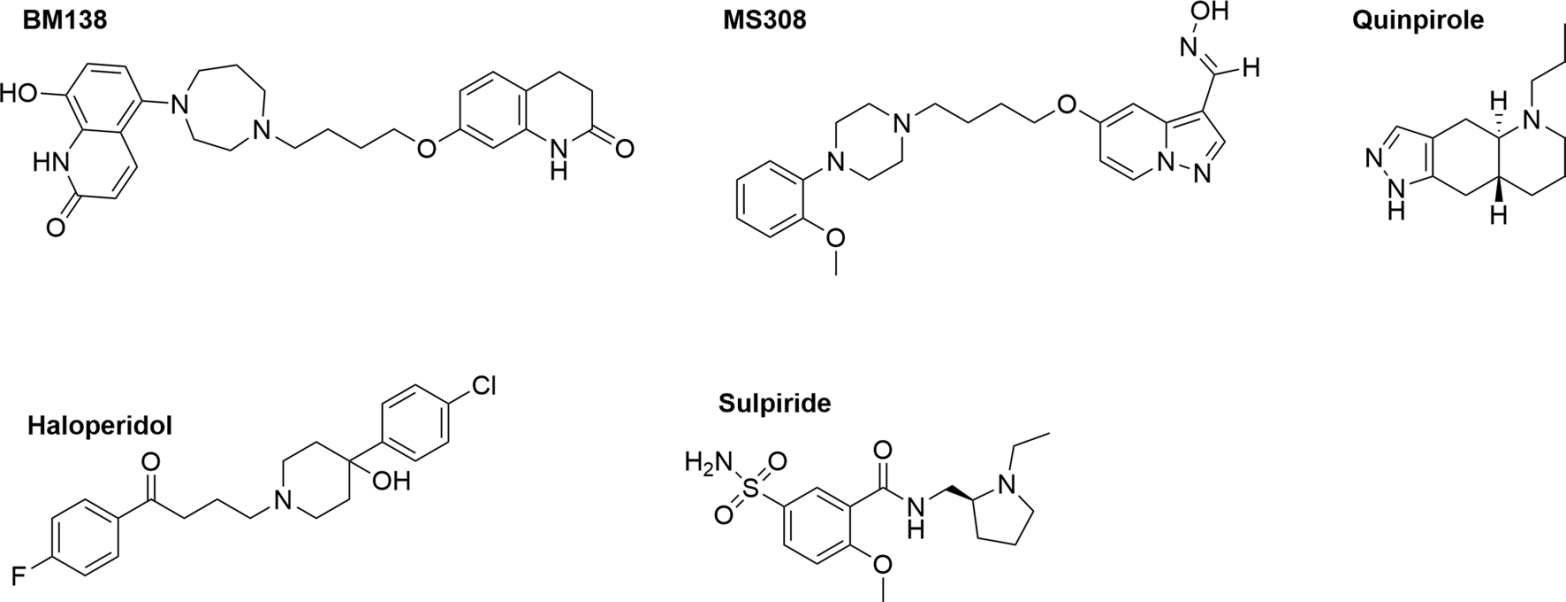
Chemical structures of the tested ligands with different functional properties. BM138 and MS308 are recently developed biased agonists, quinpirole is a balanced agonist, haloperidol and sulpiride are antagonists of D_2_R.

As representative agonists, the β-arrestin-preferring agonist BM138 (compound 13a in^21^), the G protein-selective partial agonist MS308 (compound 16c in^20^) and the balanced reference agonist quinpirole were chosen. In previously conducted BRET-biosensor assays with HEK293 cells expressing the D_2S_R, the G protein-selective partial agonist MS308 activated Gα_i1-3_ proteins with a maximum efficacy (E_max_) of 50–62% and Gα_oA/B_ proteins with a maximum efficacy (E_max_) of 77–82%, while it acted as an antagonist for the recruitment of β-arrestin-2^20^. In contrast, BM138 was a full agonist for the recruitment of β-arrestin-2 in the presence and absence of G protein-coupled receptor kinase 2 (GRK2) in an enzyme-fragment complementation assay (PathHunter, DiscoverX), while it exhibited a maximum efficacy of 55% for the activation of G proteins as characterized in a [^35^S]GTPγS binding assay^21^. The balanced full agonist quinpirole was used as a reference agonist for D_2_R activation in all assay systems^20,21^. Additionally, the clinically used drugs haloperidol and sulpiride were selected as representative antagonists for comparison. Their functional properties are well described in the literature^25,26^. Figure 1 gives an overview of the chemical structures of all tested ligands and Table 1 lists their *K*_i_ values and efficacy.

**Table 1:** *K*_i_ values and efficacy of the ligands BM138, MS308, quinpirole, haloperidol and sulpiride.

	BM138	MS308	Quinpirole	Haloperidol	(-)-Sulpiride
*K*_*i*_ (D_2S_R)	0.81 nM^21^	0.094 nM^20^	70 nM^63^	0.45 nM^25^	50.1 nM^35^
G protein	Partial agonist^21^	Partial agonist^20^	Full agonist^20,21,63^	Antagonist/inverse agonist^25,34^	Antagonist/inverse agonist^25,34,35^
β-arrestin	Full agonist^21^	Antagonist^20^	Full agonist^20,21,63^	Antagonist^20,63^	Antagonist^64^

## Results

Human embryonic kidney (HEK) 293 T cells transiently transfected with D_2S_R were selected as in vitro model because this overexpression model ensures that the receptor of interest, D_2S_R, is primarily responsible for any observed changes in the proteome. Furthermore, the receptor-activation profiles of multiple ligands have been determined in this cell type before, providing functional data for the ligands investigated in this study.

The impact of the functionally selective and balanced D_2_R ligands on the proteome was investigated after incubating D_2S_R-overexpressing HEK293T cells with the test compounds. Receptor densities were validated by radioligand binding assays conducted with membrane preparations of cells transfected within the same batch as the cells used for the proteome analysis (B_max_ 8400 ± 690 fmol/mg total protein). Finally, the quantitative changes of intracellular proteins after stimulation with the ligands were determined via shotgun liquid chromatography–tandem mass spectrometry (LC–MS/MS).

### Proteins detected in HEK293T-D_2S_R cells incubated with D_2_R ligands

**Figure 2:**
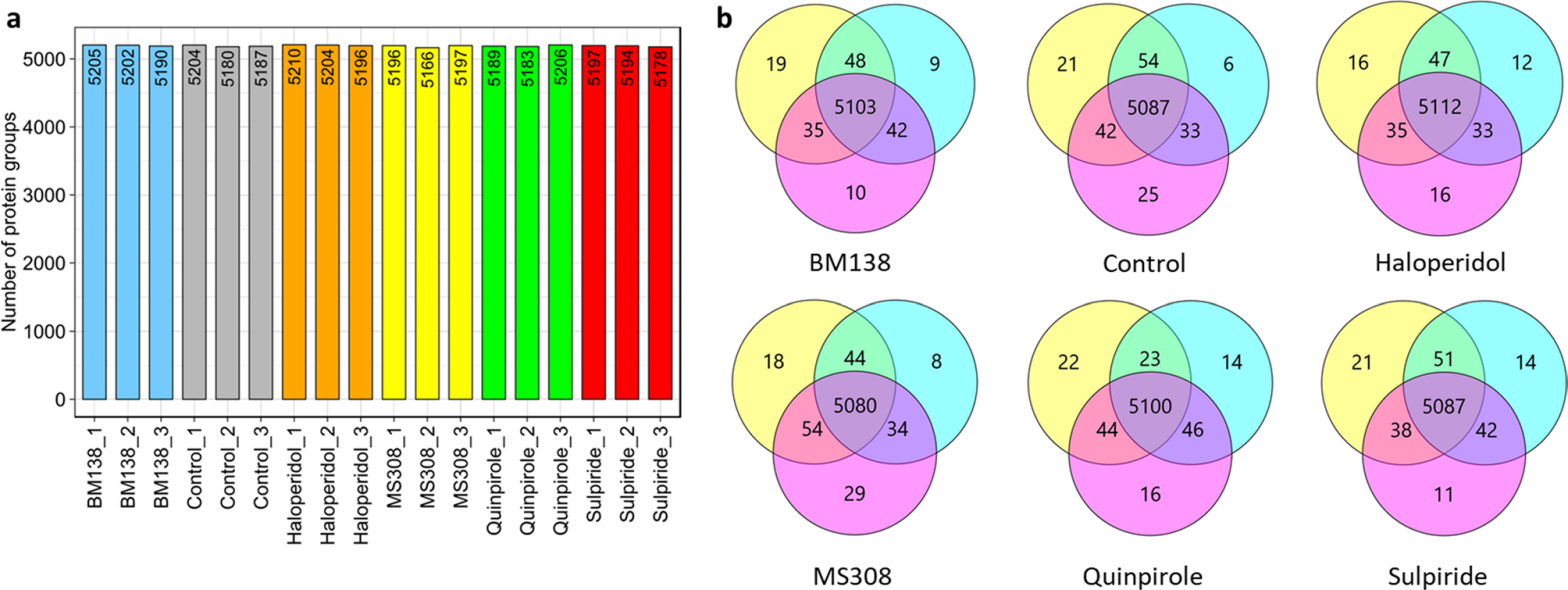
Overview of identified protein groups (PGs) of D_2S_R expressing HEK293T cells incubated with the D_2_R ligands BM138, haloperidol, MS308, quinpirole and sulpiride. (**a**) Number of PGs identified in replicates of all conditions. (**b**) Venn diagrams of PGs identified in the different replicates, shown per condition.

The proteomic analysis of whole cell lysates of HEK293T-D_2S_R cells incubated with the different D_2_R ligands identified 5290 protein groups (PGs) in total. A PG contains all proteins that could be assigned to a set of detected peptides^27^, in the present case mainly protein isoforms or different protein subunits. Similar numbers of PGs were detected in the samples (three replicates per condition, Fig. 2a). The data reproducibility was determined by comparing the PGs within each set of replicates. More than 99% of the PGs were identified in at least two of three replicates indicating good reproducibility of the experiment with only marginal random sampling, including incubation, cell harvest, sample preparation and LC–MS/MS measurement. Figure 2b shows the overlap of PGs detected in three replicates of either vehicle-treated control cells or cells incubated with one of five D_2_R ligands.

### Qualitative and quantitative differences between protein expression profiles of HEK293T-D_2S_R cells incubated with D_2_R ligands

Comparing PGs identified in the different conditions showed the greatest similarities between the cells treated with the antagonists haloperidol or sulpiride (99% of PGs overlap). However, also cells treated with different D_2_R ligand types (unbiased agonist, functionally selective agonist, antagonist) shared most of their proteome with each other and a vehicle-treated control. Ninety-five percent of the identified PGs were detected in at least two replicates of each condition.

**Figure 3:**
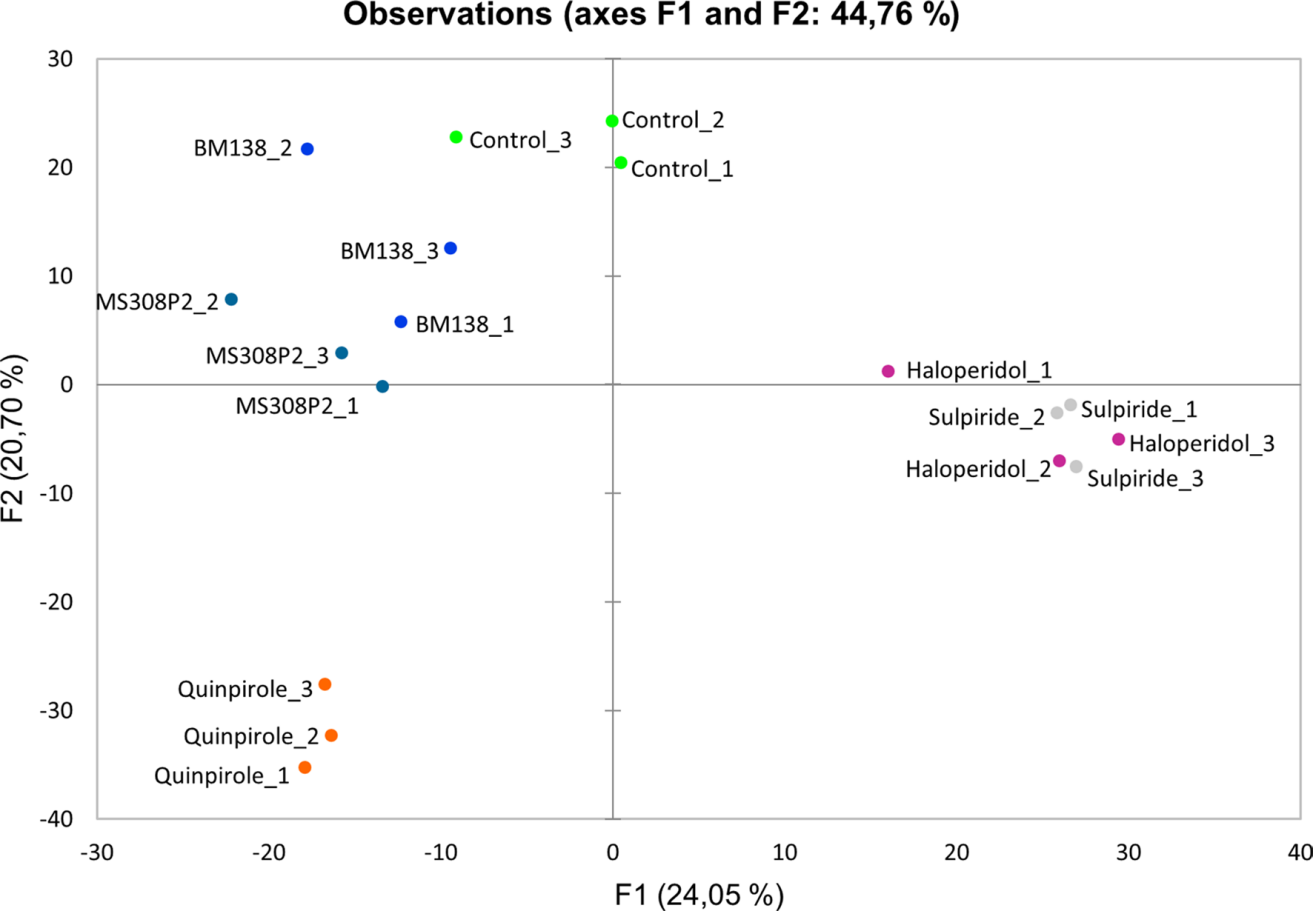
2D visualization from PCA of the proteome of vehicle-treated control and ligand-treated HEK293T-D_2s_R cells, using the normalized peak areas of 1462 differentially expressed proteins (ANOVA *p*-value < 0.05). Cells were incubated with four different classes of D_2_R ligands (β-arrestin-preferring agonist BM138, G protein-biased partial agonist MS308, unbiased agonist quinpirole, antagonists haloperidol and sulpiride). Each incubation including the control treatment was performed in triplicate.

While the differently treated cells shared most of the expressed proteins, the expression levels of these proteins differed substantially between the treatments (see Supplementary Table S1 online). Of the 5290 quantified PGs, 1462 were differentially expressed between at least two of the six conditions according to analysis of variance (ANOVA; n = 3, *p*-value < 0.05). The normalized peak areas of these 1462 differentially expressed proteins were subjected to principal component analysis (PCA) to examine whether the different conditions form clusters. As depicted in the resulting 2D observation graph (Fig. 3), all conditions formed separate clusters, with the exception of the two antagonists haloperidol and sulpiride. All samples were assembled into three major groups, one consisting of the reference full agonist quinpirole, one comprising the two antagonists haloperidol and sulpiride and one containing the two functionally selective ligands BM138 and MS308 as well as the vehicle-treated control.

**Figure 4:**
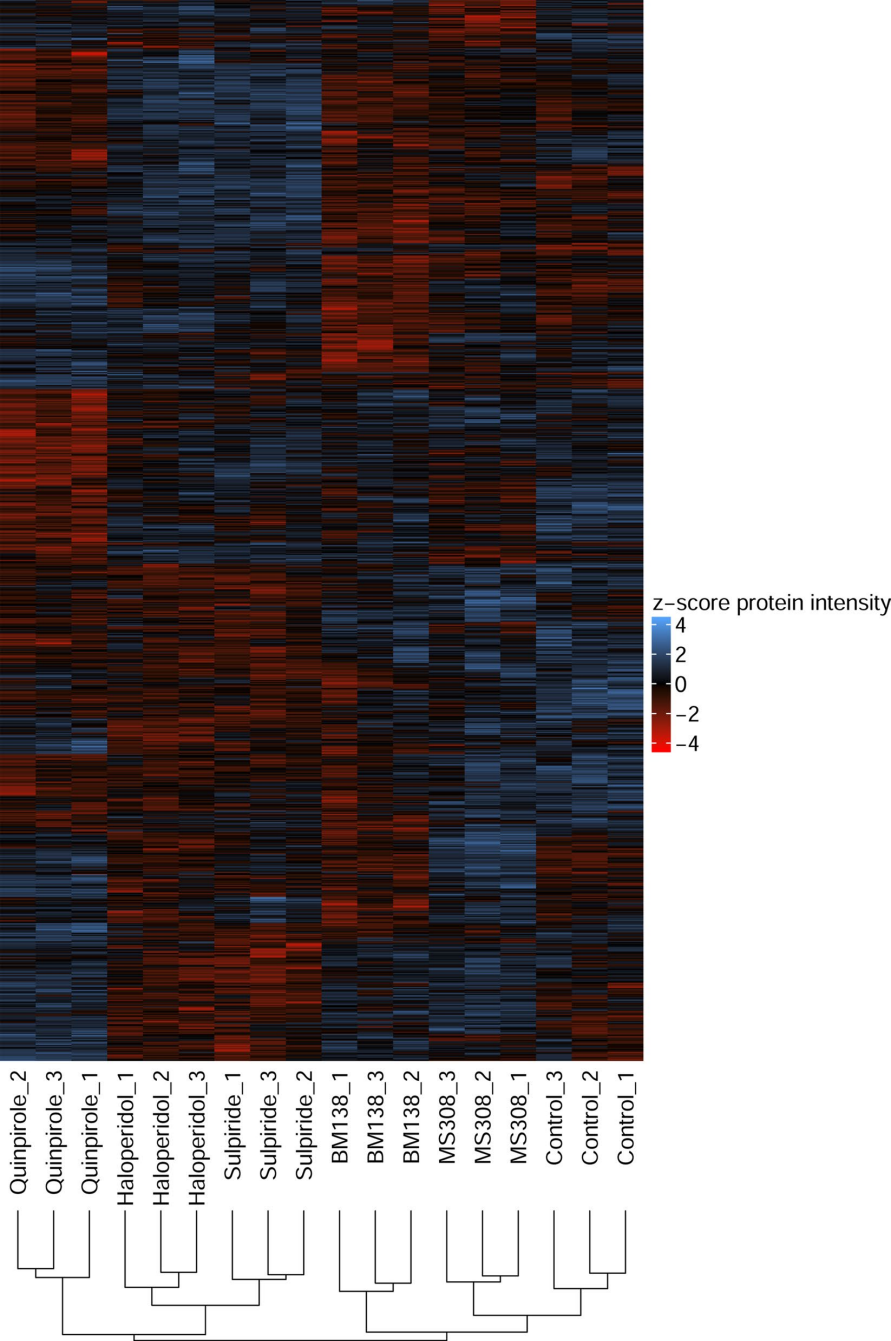
Heatmap showing the relative abundance of 1462 proteins that demonstrated differential expression between any of the conditions (ANOVA *p*-value < 0.05), color-coded based on the z-score of the proteins’ normalized peak area. Hierarchical clustering showed good separation of the different conditions.

In addition, hierarchical clustering of the samples was performed based on the normalized peak areas of differential proteins. Figure 4 shows the relative abundance of the 1462 differentially expressed PGs, represented by the z-score of the proteins’ normalized peak area. The replicates of each condition were clustered first, indicating that the acquired proteome data is suitable to distinguish between the different conditions including the two antagonists. Next, the two antagonists haloperidol and sulpiride were clustered. The vehicle-treated control was first clustered with the G protein-selective partial agonist MS308, directly followed by the β-arrestin-preferring agonist BM138. The balanced full agonist quinpirole was the last agonist to be clustered with the control. Interestingly, the unbiased agonist quinpirole clustered at first with the antagonists haloperidol and sulpiride and then with the vehicle-treated control. However, the distance between the two clustered groups (quinpirole, haloperidol and sulpiride vs. control, MS308 and BM138) is very small, indicating only marginal differences.

### Functional analysis of proteins regulated by D_2_R ligands

In PCA, factor 1 clearly separated antagonists (positive values) from agonists (negative values). The proteins exhibiting the largest positive associations with factor 1 were subjected to functional enrichment analysis. The aim was to elucidate Gene Ontology (GO) molecular functions, biological processes and cellular compartments characteristic for the treatment with the two antagonists haloperidol and sulpiride, as well as pathways derived from the databases KEGG^28,29^, Reactome and Wiki Pathways using the web-based tool g:Profiler^30^ against a background of all 1462 differential proteins. Significant hits (adjusted *p*-value ≤ 0.05) for GO biological processes are summarized in Table 2. The analysis revealed a significant enrichment of proteins regulating cellular transport (especially protein transport) as well as proteins implicated in export from cell, secretion, and exocytosis in the cells treated with the antagonists. Additionally, many proteins involved in peroxisome function and lipid modification were significantly enriched. Moreover, the analysis showed a highly significant enrichment of the cellular compartments membrane, endoplasmic reticulum and peroxisome (for the complete overview including GO cellular components and KEGG terms see Supplementary Table S2 online).

**Table 2:** GO biological processes associated with antagonist (haloperidol and sulpiride) treatment as determined by PCA.

Source	Term name	Adjusted *p*-value	Term size	Intersection size
GO:BP	Transport	2.98E−05	493	62
GO:BP	Establishment of localization	1.03E−04	506	62
GO:BP	Localization	1.16E−04	593	68
GO:BP	Lipid modification	5.56E−04	25	12
GO:BP	Export from cell	1.05E−03	158	30
GO:BP	Peroxisome organization	1.49E−03	14	9
GO:BP	Peroxisomal transport	1.49E−03	14	9
GO:BP	Protein transport	1.72E−03	285	42
GO:BP	Organic substance transport	2.06E−03	321	45
GO:BP	Amide transport	2.15E−03	287	42
GO:BP	Peptide transport	2.15E−03	287	42
GO:BP	Secretion by cell	2.42E−03	154	29
GO:BP	Protein localization	2.55E−03	371	49
GO:BP	Secretion	3.09E−03	165	30
GO:BP	Nitrogen compound transport	3.19E−03	302	43
GO:BP	Establishment of protein localization	5.12E−03	295	42
GO:BP	Vesicle-mediated transport	5.50E−03	262	39
GO:BP	Macromolecule localization	6.91E−03	394	50
GO:BP	Response to organic substance	1.48E−02	317	43
GO:BP	Protein targeting to peroxisome	1.55E−02	13	8
GO:BP	Establishment of protein localization to peroxisome	1.55E−02	13	8
GO:BP	Protein localization to peroxisome	1.55E−02	13	8
GO:BP	Cellular protein localization	1.95E−02	285	40
GO:BP	Cellular macromolecule localization	1.95E−02	285	40
GO:BP	Exocytosis	3.86E−02	117	23
GO:BP	Regulated exocytosis	4.07E−02	100	21

Proteins featuring the 10% highest positive association with factor 1 in PCA were compared to a background of all 1462 differential proteins using the web-based platform g:Profiler (*p*_adj_ ≤ 0.05).

Functional enrichment analysis of proteins with the largest negative association with factor 1 produced no significant results (*p*_adj_ > 0.05), maybe because the agonists show a wider spread around factor 2. Therefore, functional enrichment analysis was performed on proteins exhibiting a negative association with both factor 1 and 2 to elucidate functional terms associated with quinpirole treatment. All significant results (*p*_adj_ ≤ 0.05) are listed in Supplementary Table S3 online. Terms related to RNA processing, mitochondrial translation and ribosome biogenesis were significantly enriched. Enriched cellular compartments included the mitochondrial matrix as well as intracellular organelle lumen, especially nuclear lumen. Functional enrichment analysis of proteins exhibiting a negative association with factor 1 and a positive association with factor 2 showed no enrichment except for the GO cellular compartment cytosol (*p*_adj_ = 6.084 × 10^–4^). Therefore, biased agonists induced only minor specific cellular responses, which did not lead to a pronounced functional enrichment.

Gene set enrichment analysis^31^ was performed on the protein lists containing all detected proteins ordered by the fold change of proteins between treatment and the control to further elucidate the effects of agonists on the proteome. Quinpirole treatment resulted in a negative enrichment of several terms in HEK293T-D_2s_R cells compared to the control (see Table 3) with the highest negative normalized enrichment scores (NES) for synaptic vesicle cycle, regulation of neurotransmitter transport and vesicle-mediated transport in synapse. However, even though the nominal *p*-values for all terms with high NES were smaller than 0.05, the false discovery rates (FDR) were rather high. This is probably due to subtle biological differences, but can also indicate that the detected proteins are not representative of the biological question, suggesting that the acquired proteome coverage might not be sufficient to capture all effects induced by quinpirole.

**Table 3:** Functional terms downregulated by the balanced agonist quinpirole as determined by gene set enrichment analysis.

Source	Term name	Intersection size	NES	*p*-value	FDR
GOBP	SYNAPTIC VESICLE CYCLE	24	− 1.89	0.002	0.544
GOBP	REGULATION OF NEUROTRANSMITTER TRANSPORT	22	− 1.85	< 0.001	0.603
GOBP	VESICLE-MEDIATED TRANSPORT IN SYNAPSE	36	− 1.85	< 0.001	0.414
GOBP	SYNAPTIC SIGNALING	52	− 1.81	< 0.001	0.514
REACTOME	ACTIVATION OF GENE EXPRESSION BY SREBF (SREBP)	25	− 1.79	0.002	0.561
GOBP	TRANS-SYNAPTIC SIGNALING	50	− 1.79	< 0.001	0.473
REACTOME	REGULLATION OF CHOLESTEROL BIOSYNTHESIS BY SREBP (SREBF)	33	− 1.78	0.002	0.450
GOBP	ESTABLISHMENT OF SYNAPTIC VESICLE LOCALIZATION	30	− 1.77	0.002	0.452
GOBP	CALCIUM ION REGULATED EXOCYTOSIS	17	− 1.75	0.002	0.528
GOBP	CHEMICAL SYNAPTIC TRANSMISSION	49	− 1.75	< 0.001	0.482
GOBP	ANTEROGRADE TRANS-SYNAPTIC SIGNALING	49	− 1.75	< 0.001	0.454
GOBP	SYNAPTIC VESICLE TRANSPORT	30	− 1.74	0.002	0.452
GOBP	SYNAPTIC VESICLE LOCALIZATION	30	− 1.74	< 0.001	0.444
GOBP	ACTIVATION OF GTPASE ACTIVITY	32	− 1.72	< 0.001	0.487
GOBP	REGULATION OF NEUROTRANSMITTER LEVELS	60	− 1.70	< 0.001	0.584
GOBP	REGULATION OF SUBSTRATE ADHESION-DEPENDENT CELL SPREADING	18	− 1.67	0.011	0.844
GOBP	NEUROTRANSMITTER SECRETION	22	− 1.66	0.019	0.868
GOBP	REGULATION OF RESPONSE TO DRUG	15	− 1.65	0.014	0.865
GOBP	SIGNAL RELEASE FROM SYNAPSE	22	− 1.65	0.016	0.855
GOBP	SYNAPTIC VESICLE RECYCLING	15	− 1.65	0.014	0.823

The 20 functional terms with the lowest NES are shown.

Gene set enrichment analysis examining the difference between functionally selective agonists and the control showed no downregulation of terms related to synapse function for the G protein-selective partial agonist MS308. Functional terms regulated by MS308, i.e. the terms with the highest or lowest NES, belonged to rather broad terms, such as DNA processing (downregulated by MS308) or mitochondrial translation (upregulated). An overview of functional terms with the 20 highest and lowest NES for MS308 is given in Supplementary Tables S4 and S5 online. Comparison of the control with the β-arrestin-preferring agonist BM138, however, revealed a downregulation of GO biological processes such as regulation of neurotransmitter transport, vesicle docking, regulation of regulated secretory pathway, (positive) regulation of exocytosis and neuron projection guidance (see Table 4) Most strikingly, it revealed a downregulation of the dopamine receptor-mediated signaling pathway.

**Table 4:** Functional terms downregulated by the β-arrestin-preferring agonist BM138 as determined by gene set enrichment analysis.

Source	Term name	Intersection size	NES	*p*-value	FDR
GOBP	RRNA PROCESSING	148	− 1.88	< 0.001	0.334
REACTOME	RRNA MODIFICATION IN THE NUCLEUS AND CYTOSOL	50	− 1.82	0.003	0.485
GOBP	REGULATION OF NEUROTRANSMITTER TRANSPORT	22	− 1.77	0.004	0.724
GOBP	REGULATION OF REGULATED SECRETORY PATHWAY	23	− 1.75	0.002	0.768
GOBP	CELLULAR RESPONSE TO ACID CHEMICAL	27	− 1.74	0.003	0.731
GOBP	VESICLE DOCKING	23	− 1.74	0.004	0.633
GOBP	REGULATION OF EXOCYTOSIS	44	− 1.73	0.003	0.569
GOBP	RRNA METABOLIC PROCESS	162	− 1.73	< 0.001	0.545
GOBP	MATURATION OF SSU-RRNA	36	− 1.70	0.008	0.692
GOBP	RIBOSOME BIOGENESIS	198	− 1.68	< 0.001	0.774
WIKIPATHWAYS	CALCIUM REGULATION IN THE CARDIAC CELL	38	− 1.67	0.003	0.851
GOBP	POSITIVE REGULATION OF EXOCYTOSIS	23	− 1.66	0.003	0.921
GOBP	RESPONSE TO ACID CHEMICAL	33	− 1.65	0.011	0.886
PANTHER PATHWAY	DOPAMINE RECEPTOR MEDIATED SIGNALING PATHWAY	21	− 1.65	0.013	0.897
GOBP	CELLULAR RESPONSE TO AMINO ACID STIMULUS	16	− 1.64	0.019	0.852
GOBP	RIBOSOMAL SMALL SUBUNIT BIOGENESIS	53	− 1.63	0.002	0.937
GOBP	MACROMOLECULE GLYCOSYLATION	40	− 1.62	0.009	0.968
MSIGDB_C2	PID_FANCONI_PATHWAY	31	− 1.62	0.007	0.967
GOBP	NEURON PROJECTION GUIDANCE	43	− 1.61	0.003	0.956
GOBP	PROTEIN GLYCOSYLATION	40	− 1.61	0.012	0.979

The 20 functional terms with the lowest NES are shown.

## Discussion

In total, the present study identified 5290 PGs in the proteome of HEK293T-D_2S_R cells, allowing for comprehensive expression profiling. Guo et al., who tested the influence of overexpression of adenosine deaminase acting on RNA 1 (ADAR1) on the HEK293T proteome, found 1495 proteins in total in non-fractionated whole cell lysates^32^. Besides 1091 proteins that were identified in both studies, 4199 additional proteins were assigned by the present study, which also used non-fractionated whole cell lysates. Geiger et al. identified 10,504 proteins in whole cell lysates of HEK293 cells after extensive sample fractionation, which sextupled the measurement time^33^.

Comparison of the PGs detected in the different conditions revealed that cells treated with the D_2_R ligands BM138, MS308, quinpirole, haloperidol and sulpiride shared 95% of their proteome with each other and a vehicle-treated control. Since all treatments affect the same receptor in only slightly different ways, this congruence is to be expected. Even cell lines of different origin share most of their proteome with each other as shown by Geiger et al. The authors compared the proteome of eleven different cell lines (including HEK293) and found that 73% of all identified proteins were detected in all cell lines, while an average of 96% of protein identifications were shared between at least two proteomes^33^.

Presently, the two conditions with the highest concordance in PGs were haloperidol and sulpiride. The two antagonists also induced similar quantitative proteome changes, as demonstrated by PCA and hierarchical clustering based on protein intensities. In PCA, the clusters formed by these antagonists overlapped, whereas hierarchical clustering could separate the two conditions. However, they were the first conditions to cluster, showing that ligands of the same type induced the most similar proteome responses. In PCA, both haloperidol and sulpiride clustered with the control after BM138 and MS308, indicating that the antagonists had a bigger impact on the proteome than the biased agonists. Although haloperidol and sulpiride are commonly referred to as dopamine antagonists, they were previously reported to act as inverse agonists for adenylyl cyclase inhibition by the D_2_R^34,35^. In contrast to neutral antagonists, inverse agonists diminish the basal activity of a GPCR shown in the absence of agonists. Thus, these two drugs reduce the spontaneous inhibitory Gα_i/o_-activity of D_2_R below its basal level, resulting in elevated cyclic adenosine monophosphate (cAMP) levels^34^. This mechanism seems to result in a substantial regulation of the proteome, further underlining the importance to distinguish between neutral antagonists and inverse agonists.

As shown by the functional enrichment analysis of distinctive proteins for the two inverse agonists in PCA, this proteomic regulation encompasses specific cellular functions and compartments. According to g:Profiler analysis, we observed a significant (*p*_adj_ ≤ 0.05) enrichment of membrane proteins. Among 97 distinctive proteins, 62 belonged to the term integral component of membrane (*p*_adj_ = 9.88 × 10^−30^), 40 to the plasma membrane (*p*_adj_ = 6.08 × 10^−3^), 37 to the endoplasmic reticulum membrane (*p*_adj_ = 3.88 × 10^−14^) and 21 to the Golgi membrane (*p*_adj_ = 8.83 × 10^−3^). Haloperidol has been proven to alter membrane properties in vitro^36^, while sulpiride did not bind to membrane models^37^. Murata et al. reported that haloperidol but not sulpiride increased the plasma membrane permeability and fluidity in the rat brain^38^. The effects were attributed to the physical properties of haloperidol, more precisely its ability to penetrate into membranes. The present study indicates that inverse agonists may influence membrane properties also indirectly via the expression of integral membrane components, independent of the drugs’ physical properties.

The biological processes significantly regulated by haloperidol and sulpiride involved transport (*p*_adj_ = 2.98 × 10^−5^), including protein transport (*p*_adj_ = 1.72 × 10^−3^) and vesicle-mediated transport (*p*_adj_ = 5.50 × 10^−3^), indicating a positive role of inverse agonists in neurotransmitter release. This hypothesis was strengthened by the significant regulation of the terms secretion (*p*_adj_ = 3.09 × 10^−3^) and exocytosis (*p*_adj_ = 3.86 × 10^−2^). The exocytosis-related proteins characteristic for haloperidol and sulpiride included proteins important for neuron function such as calnexin and syntaxin-4. Strikingly, many of the proteins that we found for the term exocytosis were also involved in neutrophil degranulation, an important process in the acute immune response. Several studies suggested an effect of haloperidol on inflammation^39–41^, but reported inconsistent results as to whether haloperidol hinders^39^ or activates^40,41^ inflammation. Matsumoto et al. proposed that haloperidol suppresses dendritic cell-induced T helper 1 immune responses in mice^42^. The authors concluded that these effects might be mediated by D_2_R. Our results also indicate that haloperidol might regulate immune responses. However, the term neutrophil degranulation itself was not significantly enriched, and besides, an in vitro cell model was used. Therefore, this hypothesis should be tested more thoroughly.

Additionally, several significantly enriched terms were related to the peroxisome, such as peroxisome organization (*p*_adj_ = 1.49 × 10^−3^), protein targeting to peroxisome (*p*_adj_ = 1.55 × 10^−2^) and peroxisome (GO:CC *p*_adj_ = 1.78 × 10^−4^, KEGG *p*_adj_ = 9.38 × 10^−6^). The peroxisome is involved in critical metabolic processes like β-oxidation of fatty acids, biosynthesis of ether phospholipids and metabolism of reactive oxygen species^43^. Since patients with disorders in peroxisome biogenesis exhibit severe functional abnormalities in the central nervous system, an important role of peroxisomes in normal brain function has been suggested^43^. A study demonstrating the central role of peroxisomes in oligodendrocyte myelination^44^ supported the hypothesis of their crucial role for neuroprotection. In the present study, the antagonist-regulated proteins linked to the peroxisome were mostly related to the β-oxidation of fatty acids mirrored by the enriched term lipid modification (*p*_adj_ = 5.56 × 10^−4^), but also to phospholipid transport and biosynthesis of lipid species such as sphingolipids, phosphatidylinositol and phosphatidylcholine.

A comparison of the three tested agonists via hierarchical clustering revealed that the balanced agonist quinpirole had more impact on the proteome than the β-arrestin-preferring agonist BM138 and the G protein-selective partial agonist MS308. The control clustered first with MS308, then with BM138 and then with quinpirole. MS308 is an antagonist for β-arrestin recruitment and a partial agonist for G protein activation and, thus, the ligand with the lowest combined efficacy (β-arrestin and G protein) of the three tested agonists. BM138, on the other hand, is a full agonist for β-arrestin recruitment and a partial agonist for G protein activation. Therefore, the primary signaling induced by quinpirole (full agonist for both pathways) is more similar to effects caused by BM138 than by MS308. This impact seems to be mirrored by the proteome. Hierarchical clustering determined the greatest impact for quinpirole, followed by BM138 and then MS308. Therefore, the degree of influence on the proteome seems to depend on the agonist’s efficacy as well as its functional selectivity. In PCA, BM138 and MS308 formed a major group with the control, whereas quinpirole and the two inverse agonists formed separate groups. In addition to the findings of hierarchical clustering, this observation indicates that the discrimination of signaling pathways by biased ligands results in either a weaker or a more precise regulation of the proteome. It also suggests that G protein-related signaling has a stronger effect on the proteome, because BM138 (full β-arrestin and partial G protein agonist) was more similar to MS308 (partial G protein agonist) than to quinpirole (full β-arrestin and full G protein agonist).

Additionally, the present study revealed major differences in downstream effects of biased and balanced agonists. While the functional enrichment analysis of quinpirole-related proteins extracted from PCA revealed rather unspecific functional terms such as RNA processing (*p*_adj_ = 2.54 × 10^−6^), mitochondrial translation (*p*_adj_ = 1.13 × 10^−4^) and ribosome biogenesis (*p*_adj_ = 1.55 × 10^−2^), the gene set enrichment analysis provided more substantial results. Here, quinpirole showed a significant downregulation of the term synaptic signaling in comparison to the control (*p* < 0.001). The regulated terms covered vesicle-mediated transport in the synapse (*p* < 0.001) as well as regulation of neurotransmitter transport (*p* < 0.001), synaptic signaling (*p* < 0.001) and calcium ion regulated exocytosis (*p* = 0.002).The downregulation suggests that D_2S_R acts as dopamine autoreceptor providing feedback inhibition to regulate cell firing and dopamine release^45^. It has been shown that the short isoform of D_2_R is mostly involved in autoreceptor functions in vivo^46,47^. The present study also hints towards a role of D_2S_R as an autoreceptor, because several proteins necessary for synaptic signaling were downregulated by balanced D_2S_R activation. In vivo studies demonstrated adverse effects of quinpirole on synaptic signaling; quinpirole treatment, i.a., inhibited currents evoked by *N*-methyl-d-aspartate (NMDA)-type post-synaptic glutamate receptors in neurons derived from the prefrontal cortex^48^ and the hippocampus^49^. The present results support these observations. However, the transferability of observations in an in vitro cell model to in vivo effects is limited.

In contrast to the balanced agonist quinpirole, the (partly) biased agonists BM138 and MS308 had a weaker influence on cellular functions. PCA did not show any enriched GO terms among their characteristic proteins except for the cellular compartment cytosol. Gene set enrichment analysis provided more information about the cellular effects of BM138 and MS308 and revealed that the G protein-selective partial agonist MS308 regulated rather broad functional terms such as mitochondrial translation and nucleosome assembly. However, BM138, the ligand showing a full agonism of β-arrestin recruitment and partial agonism for G protein activation, downregulated functional terms such as the regulation of neurotransmitter transport, vesicle docking and regulation of exocytosis. Therefore, BM138 also induced functions characteristic for quinpirole, but to a lesser degree. The NES for regulation of neurotransmitter transport was − 1.85 for quinpirole and − 1.77 for BM138. Moreover, the term synaptic signaling itself was not regulated by BM138, also indicating a weaker effect compared to quinpirole. In drug development, selective targeting of β-arrestin-2 downstream of dopamine receptors was proposed to relieve l-DOPA-induced dyskinesia in the treatment of Parkinson´s disease, since this side effect was associated with the overactivation of G protein-mediated signaling^24^. In the present study, BM138 showed a lower impact on D_2_R-induced cellular functions than quinpirole, hinting towards a more precise regulation of the proteome that might reduce side effects. Since BM138 shows the same efficacy for β-arrestin as quinpirole, but a lesser efficacy for G protein activation, the increased G protein-mediated signaling by quinpirole is probably responsible for the observed different cellular effects. However, further studies are necessary to clarify whether these effects are beneficial or detrimental in vivo. Interestingly, MS308 did not regulate any synapse-related terms. MS308 is a partial agonist for G protein activation like BM138, but does not show any β-arrestin-2 recruitment. Beaulieu et al. reported β-arrestin-2 signaling downstream of D_2_R through a G protein-independent Akt/GSK3 pathway in vivo, which resulted in dopamine-dependent behaviors^50^. The present study supports the important role of β-arrestin-mediated signaling in D_2_R function.

However, β-arrestin-linked pathways and classical G protein signaling involve temporally distinct signaling cascades^1^, with β-arrestin-based signaling following G protein pathways in D_2_R signaling^51^. Since we presently tested only one stimulation time (6 h), further experiments are important to investigate time courses for a full characterization of ligand-induced proteome changes. Additionally, more representatives per ligand type (G protein-biased agonists, arrestin-biased ligands such as UNC9994^6^ and balanced agonists such as ergoline, aporphine or aminotetraline) should be assessed to confirm whether the observed effects can be attributed to all ligands of the same type or specifically to one single drug. Furthermore, the transferability of the obtained results to other cell and animal models should be evaluated. These models, however, may not be able to elucidate the individual role of the receptors, because multiple GPCRs are co-expressed in vivo. Thus, the accurate assessment of the ligands’ functional characteristics is difficult. Ligands are frequently not fully selective for a single GPCR, but can also bind to other GPCRs. Because HEK293 cells are known to express multiple GPCRs^52^, the changes observed in this study might also not be fully assignable to D_2S_R stimulation. However, the endogenous levels of GPCRs in HEK293 cells are rather low^52^. Hence, we suggest that the influence of the overexpressed D_2S_R should predominate. This is in agreement with negligible influences of quinpirole and haloperidol on the concentration of the second messenger inositol monophosphate (IP), that we found in HEK293T cells in the absence of D_2S_R overexpression (Supplementary Figure S1).

In summary, it could be shown that the activation of D_2S_R with different ligand classes is mirrored on the proteome level, with more pronounced differences in the quantitative proteome. The degree of influence on the proteome and, thereby, cellular function seems to be dependent on the ligand’s efficacy for both G protein- and β-arrestin-mediated signaling. In this respect, G protein-mediated signaling seems to have a stronger impact on the cellular protein composition. However, β-arrestin-mediated signaling appears to play a more important role in D_2S_R-mediated function. Balanced D_2S_R activation led to a downregulation of proteins related to synaptic signaling, supporting the role of D_2S_R as a dopamine autoreceptor. Additionally, strong effects of inverse agonists on cellular functions could be observed, highlighting the importance to distinguish between inverse agonists and antagonists.

## Methods

### Cell culture and incubation conditions

HEK293T cells were maintained in DMEM-F12 medium supplemented with 10% fetal bovine serum, 100 I.U./mL penicillin G, 100 µg/mL streptomycin and 2 mM glutamine at 37 °C and 5% CO_2_ in a humidified atmosphere. At 80% confluency in a 15 cm diameter plate, the cells (passage 5) were transfected with D_2S_R via the Mirus *Trans*IT293 transfection reagent according to the manufacturer’s instructions (Mirus Bio LLC). The medium was changed 24 h after transfection. After further 24 h, the cells (passage 5, from the same transfection batch, on three culture plates each) were incubated for 6 h with the respective D_2_R ligands or the control testing each condition simultaneously in triplicate. The ligands BM138 and MS308 were synthesized as reported previously^20,21^. The incubation solutions contained either 3 µM BM138, 1 µM haloperidol, 1 µM MS308, 3 µM quinpirole, 10 µM sulpiride or, as vehicle-treated control, no agent in 0.01% DMSO in complete growth medium each. The chosen concentrations represent the saturation concentrations for each ligand to ensure full receptor occupancy. No phenotypic changes were observed in the cells via light microscopy throughout the process. After removing the incubation medium, the cells were detached with versene followed by two wash cycles with ice-cold phosphate-buffered saline (PBS) and centrifugation at 200×*g*_E_ for 6 min at room temperature. After removing PBS, the cells were lysed with five freeze–thaw cycles in liquid nitrogen and proteins were extracted with 3.4 mL of ultrapure water. The aqueous protein extract was freeze-dried for storage until analysis. In one aliquot of each aqueous protein extract, the protein content was determined with the bicinchoninic acid assay (Pierce BCA protein assay kit).

### Membrane preparation and radioligand saturation binding

Receptor expression levels were determined following previous protocols^53^. In brief, HEK293T cells were transfected as described above and the growth medium was removed 48 h after transfection. The cells were washed once with 5 mL of ice-cold PBS, and subsequently detached by rinsing with harvest buffer (10 mM Tris-HCl, 0.5 mM EDTA, 5.4 mM KCl, 140 mM NaCl, pH 7.4). After centrifugation (8 min, 200 × *g*_E_) the pellet was resuspended in 10 mL of ice-cold homogenate buffer (50 mM Tris-HCl, 5 mM EDTA, 1.5 mM CaCl_2_, 5 mM MgCl_2_, 5 mM KCl, 120 mM NaCl, pH 7.4) and lysed with an ultraturrax. After ultracentrifugation (50,000 × *g*_E_, 20 min) the membranes were resuspended in binding buffer (50 mM Tris–HCl, 1 mM EDTA, 5 mM MgCl_2_, 100 µg/mL bacitracin, 5 µg/mL soybean trypsin inhibitor, pH 7.4) and homogenized with a glass-Teflon homogenizer. The membrane preparations were shock-frozen in liquid nitrogen and stored at − 80 °C until usage. The protein concentration was determined with the method of Lowry^54^ applying bovine serum albumin as standard. Saturation binding experiments were performed in 96-well format with [^3^H]spiperone (Perkin Elmer, specific activity 68 Ci/mmol) as the radioligand. Total binding was determined by incubation of the membranes with varying concentrations of [^3^H]spiperone (0.05 nM to 2.00 nM) in binding buffer (total protein concentration 30 µg/mL). Non-specific binding was determined in the presence of 10 µM haloperidol. After 1 h of incubation at 37 °C, the reaction was terminated by filtration through GF/B filters soaked with 0.3% polyethyleneimine solution, followed by five washes with ice-cold buffer (50 mM Tris, 120 mM NaCl, pH 7.4). The filters were air-dried at 60 °C for 3 h, sealed with scintillation wax and the bound radioactivity was determined with a Microbeta counter (Perkin Elmer). Total, non-specific, and specific binding were analyzed employing the algorithms for one-site saturation binding implemented in PRISM 8.0 (GraphPad) to determine the equilibrium dissociation constant (K_D_) of the radioligand and the receptor expression level (B_max_).

### Accumulation of inositol monophosphate (IP) as functional assay for G protein-mediated signaling

Determination of GPCR stimulation on the level of second messenger accumulation was performed applying the IP-One HTRF assay (Cisbio) according to the manufacturer’s protocol and as described previously^55^. In brief, HEK293T cells were grown to a confluence of ~ 70% and transfected with the cDNAs of the Gα_q_ protein or the hybrid G proteins Gα_qs_ or Gα_qi_, respectively (hybrid G proteins are Gα_q_ proteins with the last five amino acids at the C-terminus replaced by the corresponding sequence of Gα_s_ or Gα_i_, respectively; gift from The J. David Gladstone Institutes, San Francisco, CA)^56^, applying the Mirus TransIT-293 transfection reagent (Peqlab). After one day, cells were detached from the culture dish with Versene (Life Technologies), seeded into black 384-well plates (10,000 cells/well) (Greiner Bio-One) and maintained for 24 h at 37 °C. The effect of quinpirole or haloperidol on any endogenously expressed GPCR was determined by incubating the test compounds (final range of concentration from 1 pM up to 10 μM) in duplicates for 90 min at 37 °C. Incubation was stopped by addition of the detection reagents (IP1-d2 conjugate and Anti-IP1cryptate TB conjugate, each dissolved in lysis buffer) followed by incubation for further 60 min at room temperature. Time-resolved fluorescence resonance energy transfer (TR-FRET) was measured using the Clariostar plate reader (BMG Labtech) applying the filter sets for emission at 620 ± 10 nm and 665 ± 10 nm, respectively. Two to four individual experiments were performed with each concentration in duplicate and the corresponding mean raw data (ratio of emission at 665 nm divided by emission at 620 nm) were compared to an IP standard (10 pM to 100 μM) concentration–response curve measured in the absence of any cells.

### Sample preparation for untargeted LC–MS/MS measurement

Each freeze-dried protein extract was solved in 100 µL of 50% 2,2,2-trifluoroethanol in 100 mM ammonium bicarbonate (ABC) buffer. The volume equivalent of 2 mg protein was aliquoted and prepared for untargeted LC–MS/MS measurement as described previously with minor modifications^57^. Shortly, proteins were denatured at 60 °C for 2 h and then reduced using 5 mM dithiothreitol at 60 °C for 30 min. Free cysteines were carbamidomethylated by incubation with 25 mM iodoacetamide for 30 min in the dark at room temperature. Samples were diluted five times (v/v) with freshly prepared 100 mM ABC buffer (pH 8.0) and calcium chloride was added to a final concentration of 2 mM. Afterwards, the samples were digested overnight with Trypsin/Lys-C Mix at 37 °C (enzyme/total protein 3:2000). Formic acid (FA) was added to a final concentration of 1% (v/v) to stop the digestion. Lastly, the samples were centrifuged for 10 min at 12,000 × *g* and 4 °C. The dried supernatants were subjected to C18 purification as described previously with minor modifications^15^. Briefly, conventional 200 µL-pipette tips were packed with five layers of 3 M Empore disk C18 material. After priming with 20 µL of methanol followed by 20 µL of 0.1% trifluoroacetic acid (TFA) in acetonitrile (ACN), the pipette tips were equilibrated with two steps of 20 µL 0.1% TFA in LC–MS grade water. Samples solved in 200 µL of 0.1% TFA were bound in two steps and washed twice with 20 µL of 0.1% FA each. Lastly, the peptides were eluted with two steps of 40 µL of 80% ACN/0.1% FA. The eluted samples were concentrated by vacuum centrifugation and then reconstituted in 125 µL of 0.1% FA in LC–MS grade water. The peptide concentrations of all samples were determined with a NanoDrop spectrophotometer (Thermo Scientific).

### LC–MS/MS measurement

A QExactive HF mass spectrometer coupled to an Easy-Spray nano-electrospray ionization source connected to an EASY-nLC 1000 nano-flow UPLC system (Thermo Scientific) was used for sample analysis. Peptides (2 µg) were loaded onto a two-column setup consisting of a 2 cm Acclaim PepMap 10 column (id 75 µm, 3 µm, 100 Å) as precolumn and a 50 cm EASY-Spray PepMap RSLC C18 column (id 75 μm, 2 μm, 100 Å) heated to 45 °C as analytical column. The flow rate was constantly set to 250 nL/min and the gradient started with 100% eluent A (0.1% (v/v) FA). Eluent B (0.1% (v/v) FA in ACN) was increased to 6% in 5 min, then to 24% in 200 min and finally to 48% in 40 min. Afterwards, eluent B was raised to 95% in 10 min and the system was rinsed for further 10 min. Data-dependent MS analyses were run in a positive top-25 mode. MS1-spectra were acquired for a mass range of *m/z* 350–1800 at a resolution of 120,000, with an automatic gain control (AGC) target of 3 × 10^6^ and 240 ms maximum fill time. The dependent MS/MS spectra were acquired at a resolution of 30,000, with an AGC target of 2 × 10^5^ and 55 ms maximum fill time. The isolation window width was set to 1.4 m*/z*, the isolation offset to 0.2 m*/z* and the intensity threshold to 1.8 × 10^3^. Dynamic exclusion was set to 60 s. A normalized collision energy of 27% was used and charge states of 1, 6, 7 and 8 as well as unassigned charge states were excluded.

### Data evaluation

The raw MS files were subjected to a protein-sequence database search via MaxQuant (version 1.5.8.3)^27^ using the FASTA-formatted uniprot entries for *Homo sapiens* with isoforms (version 2015.01.27, number of entries 42,041). The main search peptide tolerance was set to ± 10 ppm and a maximum of two missed cleavages was allowed. Carbamidomethyl (C) was set as fixed modification; oxidation (M) and acetylation (protein N-term) were chosen as variable modifications. The false discovery rate was kept at 1% using a target-decoy strategy. PGs were considered identified in single samples when at least one razor or unique peptide was detected. Razor peptides are peptides that are not unique and are therefore assigned with priority to the PG with the most identified peptides^58^. For the comparison between conditions, PGs were considered identified when they were detected in at least two of the three replicates of the condition by at least one razor or unique peptide. All Venn diagrams were created with VennDIS^59^. For quantification, the PG intensities were first modified according to Wojtowicz et al.^60^ and then logarithmized (log_2_) to transform the data to a normal distribution.

### Statistical and functional analysis

All subsequent data evaluation was done with R (version 3.5.3, complex heatmap package^61^) with the exception of PCA, which was performed using the XLSTAT software (Addinsoft, Paris, France). For statistical testing, hierarchical clustering, and PCA, missing values were imputed from the lower distribution as described by Tyanova et al.^58^. Statistical significance was determined by ANOVA (*p* < 0.05, six groups, n = 3) and hierarchical clustering of treatments was performed on PG intensities after z-score normalization using Euclidean distances between averages. PCA was completed applying Pearson’s correlation matrix to the intensities of all differentially expressed PGs without z-score normalization, using the gene names derived from MaxQuant as PG identifiers. The eigenvector was used as a measure for the protein’s contribution to a factor. Proteins showing a certain contribution to a factor (e.g. for quinpirole, a negative eigenvector for factor 1 and a negative eigenvector for factor 2) were then submitted to functional enrichment analysis using the web-based tool g:Profiler^30^, with all differential proteins as background and GO, KEGG^28,29^, Reactome and WikiPathways as data sources. The results of functional enrichment analysis are significantly enriched annotation terms (i.e. GO terms) within a subset of submitted proteins. For multiple-testing correction, the p-value was adjusted using the g:SCS algorithm. Gene set enrichment analysis was carried out using the GSEA software^31^ on pre-ranked lists containing all proteins (identified by gene names) ordered by their fold change between a treatment and the control. As data source, a GMT file obtained from http://baderlab.org/GeneSets (version 2019.04.01) was used^62^. It contained all gene sets from GO biological process, excluding electronic annotations, and all pathway resources (including, i.a., KEGG^28,29^, Reactome, and Panther). Analysis was carried out using all gene sets between 15 and 200 genes and 1000 permutations.

## Supplementary Information

Supplementary Table 1.

Supplementary Information.

## Data Availability

The raw MS data associated with this manuscript was submitted to the Mass Spectrometry Interactive Virtual Environment (ftp://MSV000086129@massive.ucsd.edu) and is available under ftp://massive.ucsd.edu/MSV000086129/.
