# Supplementary material for: Functionally selective activation of the dopamine receptor D2 is mirrored by the protein expression profiles
Source: Sci Rep. 2021 Feb 10;11:3501. doi: 10.1038/s41598-021-83038-x (PMC7875989; doi:10.1038/s41598-021-83038-x)
Supplement: Supplementary file 2 — Supplementary Information. [file 41598_2021_83038_MOESM2_ESM.pdf]

Supplementary Information

Functionally selective activation of the dopamine receptor D_2 is mirrored by the protein expression profiles

Deborah Wenk[1], Vladimir Ignatchenko[2], Andrew Macklin[2], Harald Hübner[3], Peter Gmeiner[3], Dorothée Weikert[3], Monika Pischetsrieder[1], Thomas Kislinger[2,4]*

[1]Food Chemistry, Department of Chemistry and Pharmacy, Friedrich-Alexander Universität Erlangen-Nürnberg (FAU), Nikolaus-Fiebiger-Str. 10, 91058 Erlangen, Germany

[2]Princess Margaret Cancer Centre, University Health Network, 101 College Street, Toronto, ON, Canada

[3]Medicinal Chemistry, Department of Chemistry and Pharmacy, Friedrich-Alexander Universität Erlangen-Nürnberg (FAU), Nikolaus-Fiebiger-Str. 10, 91058 Erlangen, Germany

[4]University of Toronto, Department of Medical Biophysics, 101 College Street, Toronto, ON, Canada

*Correspondence to: monika.pischetsrieder@fau.de

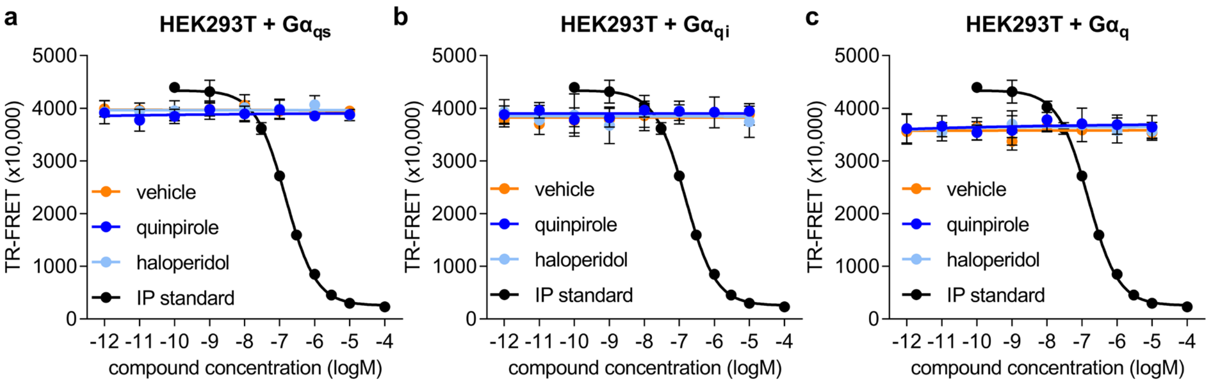

Supplementary Figure S1. G protein activation in HEK293T cells in the absence of overexpressed receptors. The contribution of endogenously expressed GPCRs to the observed signaling profiles in HEK293T cells was investigated on the level of the second messenger inositol monophosphate (IP) employing the IP-One HTRF assay (Cisbio). HEK293T cells were transfected with (**a**) the $G\alpha_q$ subunit or (**b**) the hybrid G protein subunits $G\alpha_{qs}$ or (**c**) $G\alpha_{qi}$ [1] to detect activation of $G\alpha_q$-, $G\alpha_s$-, or $G\alpha_i$-coupled GPCRs under the same assay conditions. Independent from the transfected Gα subunit, neither quinpirole nor haloperidol induced substantial changes in the cellular IP level, pointing towards a negligible contribution of endogenously expressed GPCRs to the overall signaling profile observed in $D_{2S}R$ overexpressing cells. The data show the mean ± standard deviation of n = 4 independent experiments for quinpirole and haloperidol and n = 2 for vehicle-treated cells. For comparison, a representative concentration-response curve obtained with an IP standard (in duplicates) is shown in panels (a-c).

[1] Broach, J. R. & Thorner, J. High-throughput screening for drug discovery. *Nature* **384** (suppl.), 14-16 doi:10.1038/384014a0 (1996).

Table S2. Functional terms associated with antagonist (haloperidol and sulpiride) treatment as determined by PCA. Proteins showing the 10% highest positive association with factor 1 in PCA were compared to a background of all 1462 differential proteins using the web-based platform g:Profiler[2] ($p_{adj} \leq 0.05$, query size 97).

Source	Term name	Adjusted p-value	Term size	Intersection size
GO:BP	transport	2.98E-05	493	62
GO:BP	establishment of localization	1.03E-04	506	62
GO:BP	localization	1.16E-04	593	68
GO:BP	lipid modification	5.56E-04	25	12
GO:BP	export from cell	1.05E-03	158	30
GO:BP	peroxisome organization	1.49E-03	14	9
GO:BP	peroxisomal transport	1.49E-03	14	9
GO:BP	protein transport	1.72E-03	285	42
GO:BP	organic substance transport	2.06E-03	321	45
GO:BP	amide transport	2.15E-03	287	42
GO:BP	peptide transport	2.15E-03	287	42
GO:BP	secretion by cell	2.42E-03	154	29
GO:BP	protein localization	2.55E-03	371	49
GO:BP	secretion	3.09E-03	165	30
GO:BP	nitrogen compound transport	3.19E-03	302	43
GO:BP	establishment of protein localization	5.12E-03	295	42
GO:BP	vesicle-mediated transport	5.50E-03	262	39
GO:BP	macromolecule localization	6.91E-03	394	50
GO:BP	response to organic substance	1.48E-02	317	43
GO:BP	protein targeting to peroxisome	1.55E-02	13	8
GO:BP	establishment of protein localization to peroxisome	1.55E-02	13	8
GO:BP	protein localization to peroxisome	1.55E-02	13	8
GO:BP	cellular protein localization	1.95E-02	285	40
GO:BP	cellular macromolecule localization	1.95E-02	285	40
GO:BP	exocytosis	3.86E-02	117	23
GO:BP	regulated exocytosis	4.07E-02	100	21
GO:CC	integral component of membrane	9.88E-30	191	62
GO:CC	intrinsic component of membrane	2.91E-29	194	62
GO:CC	organelle membrane	1.47E-19	359	70
GO:CC	endoplasmic reticulum	8.48E-17	190	50
GO:CC	endomembrane system	1.96E-16	451	74
GO:CC	nuclear outer membrane-endoplasmic reticulum membrane network	7.48E-15	115	38
GO:CC	endoplasmic reticulum membrane	3.88E-14	113	37
GO:CC	bounding membrane of organelle	5.62E-13	218	49
GO:CC	membrane	5.88E-12	704	85
GO:CC	whole membrane	9.00E-10	190	42
GO:CC	integral component of plasma membrane	1.96E-08	40	19
GO:CC	intrinsic component of plasma membrane	3.46E-08	41	19
GO:CC	vesicle membrane	7.46E-06	92	25
GO:CC	cytoplasmic vesicle membrane	2.27E-05	89	24
GO:CC	microbody	1.78E-04	19	11
GO:CC	peroxisome	1.78E-04	19	11
GO:CC	intracellular vesicle	2.04E-04	267	42
GO:CC	cytoplasmic vesicle	2.04E-04	267	42

GO:CC	membrane protein complex	2.57E-04	107	25
GO:CC	vesicle	4.67E-04	416	54
GO:CC	Golgi apparatus	1.58E-03	170	31
GO:CC	secretory granule membrane	1.58E-03	32	13
GO:CC	microbody membrane	1.92E-03	8	7
GO:CC	peroxisomal membrane	1.92E-03	8	7
GO:CC	plasma membrane	6.08E-03	274	40
GO:CC	Golgi membrane	8.82E-03	92	21
GO:CC	lytic vacuole membrane	8.92E-03	55	16
GO:CC	lysosomal membrane	8.92E-03	55	16
GO:CC	cell periphery	9.06E-03	289	41
GO:CC	integral component of endoplasmic reticulum membrane	1.15E-02	21	10
GO:CC	lytic vacuole	1.32E-02	86	20
GO:CC	lysosome	1.32E-02	86	20
GO:CC	microbody lumen	1.55E-02	13	8
GO:CC	peroxisomal matrix	1.55E-02	13	8
GO:CC	intrinsic component of endoplasmic reticulum membrane	1.99E-02	22	10
GO:CC	transport vesicle	2.03E-02	58	16
GO:CC	membrane-bounded organelle	2.20E-02	1246	97
GO:CC	vacuole	2.34E-02	97	21
GO:CC	integral component of organelle membrane	2.41E-02	33	12
GO:CC	vacuolar membrane	2.63E-02	59	16
KEGG[3,4]	Peroxisome	9.38E-06	10	9

[2] Raudvere, U. *et al.* g:Profiler: a web server for functional enrichment analysis and conversions of gene lists (2019 update). *Nucleic Acids Res.* **47**, W191-W198, doi:10.1093/nar/gkz369 (2019).

[3] Kanehisa, M. & Goto, S. KEGG: kyoto encyclopedia of genes and genomes. *Nucleic Acids Res.* **28**, 27-30, doi:10.1093/nar/28.1.27 (2000).

[4] Kanehisa, M. Toward understanding the origin and evolution of cellular organisms. *Protein Sci.* **28**, 1947-1951, doi:10.1002/pro.3715 (2019).

Table S3. Functional terms associated with quinpirole treatment as determined by PCA. All proteins showing a negative association with both factor 1 and factor 2 in PCA were compared to a background of all 1462 differential proteins using the web-based platform g:Profiler ($p_{adj} \leq 0.05$, query size 334).

Source	Term name	Adjusted p-value	Term size	Intersection size
GO:MF	RNA binding	8.77E-03	421	136
GO:BP	RNA processing	2.54E-06	231	94
GO:BP	mitochondrial translation	1.13E-04	45	29
GO:BP	mitochondrial gene expression	2.48E-04	51	31
GO:BP	ncRNA processing	6.81E-04	94	46
GO:BP	rRNA processing	3.74E-03	58	32
GO:BP	rRNA metabolic process	6.46E-03	59	32
GO:BP	mitochondrial translational elongation	1.02E-02	34	22
GO:BP	ribosome biogenesis	1.55E-02	72	36
GO:BP	gene expression	2.02E-02	626	185
GO:BP	mitochondrial translational termination	2.98E-02	33	21
GO:BP	ncRNA metabolic process	3.73E-02	117	50
GO:BP	ribonucleoprotein complex biogenesis	4.87E-02	102	45
GO:CC	mitochondrial matrix	3.02E-07	115	59
GO:CC	membrane-enclosed lumen	6.81E-07	776	233
GO:CC	organelle lumen	6.81E-07	776	233
GO:CC	intracellular organelle lumen	6.81E-07	776	233
GO:CC	ribonucleoprotein complex	1.32E-04	177	74
GO:CC	mitochondrial protein complex	9.72E-04	43	27
GO:CC	organelle inner membrane	1.71E-03	54	31
GO:CC	mitochondrial membrane	4.12E-03	72	37
GO:CC	mitochondrial inner membrane	6.60E-03	51	29
GO:CC	nuclear lumen	9.40E-03	600	180
GO:CC	mitochondrion	2.04E-02	216	80
GO:CC	mitochondrial ribosome	2.41E-02	28	19
GO:CC	organellar ribosome	2.41E-02	28	19
REAC	Mitochondrial translation elongation	1.02E-02	34	22
REAC	Mitochondrial translation initiation	1.37E-02	32	21
REAC	Mitochondrial translation	2.16E-02	35	22
REAC	Mitochondrial translation termination	2.98E-02	33	21

Table S4. Functional terms upregulated by MS308 as determined by gene set enrichment analysis[5].

The table lists the 20 functional terms with the highest normalized enrichment score (NES).

Source	Term name	Size	NES	p-value	FDR
GOBP	CELL-SUBSTRATE ADHESION	28	1.85	0.004	0.457
REACTOME	MITOCHONDRIAL TRANSLATION	76	1.85	< 0.001	0.261
GOBP	MITOCHONDRIAL GENE EXPRESSION	107	1.84	< 0.001	0.196
GOBP	MITOCHONDRIAL TRANSLATION	92	1.82	0.002	0.197
REACTOME	MITOCHONDRIAL TRANSLATION TERMINATION	71	1.80	< 0.001	0.226
GOBP	CELL-MATRIX ADHESION	20	1.80	< 0.001	0.196
GOBP	MITOCHONDRIAL TRANSLATIONAL ELONGATION	73	1.80	< 0.001	0.177
REACTOME	MITOCHONDRIAL TRANSLATION ELONGATION	72	1.80	< 0.001	0.161
REACTOME	MITOCHONDRIAL TRANSLATION INITIATION	71	1.78	< 0.001	0.179
GOBP	MITOCHONDRIAL TRANSLATIONAL TERMINATION	72	1.78	0.002	0.179
GOBP	TRANSLATIONAL TERMINATION	78	1.77	< 0.001	0.166
GOBP	TRANSLATIONAL ELONGATION	87	1.77	< 0.001	0.173
REACTOME	RUNX1 INTERACTS WITH CO-FACTORS WHOSE PRECISE EFFECT ON RUNX1 TARGETS IS NOT KNOWN	24	1.75	0.006	0.196
GOBP	NUCLEOSIDE BIOSYNTHETIC PROCESS	15	1.73	< 0.001	0.243
GOBP	CELL ADHESION	101	1.73	< 0.001	0.250
GOBP	POSITIVE REGULATION OF PROTEIN LOCALIZATION TO CELL PERIPHERY	21	1.73	0.012	0.234
WIKIPATHWAYS	ARRHYTHMOGENIC RIGHT VENTRICULAR CARDIOMYOPATHY	15	1.71	0.006	0.272
WIKIPATHWAYS	PATHWAYS AFFECTED IN ADENOID CYSTIC CARCINOMA	31	1.71	0.002	0.278
GOBP	POSITIVE REGULATION OF PROTEIN LOCALIZATION TO PLASMA MEMBRANE	17	1.70	0.004	0.272
GOBP	BIOLOGICAL ADHESION	104	1.70	0.002	0.266

[5] Subramanian, A. *et al.* Gene set enrichment analysis: a knowledge-based approach for interpreting genome-wide expression profiles. *Proc. Natl. Acad. Sci. U. S. A.* **102**, 15545-15550, doi:10.1073/pnas.0506580102 (2005).

Table S5. Functional terms downregulated by MS308 as determined by gene set enrichment analysis.

The table lists the 20 functional terms with the lowest normalized enrichment score (NES).

Source	Term name	Size	NES	p-value	FDR
REACTOME	DEPOSITION OF NEW CENPA-CONTAINING NUCLEOSOMES AT THE CENTROMERE	18	−1.89	0.002	0.463
REACTOME	RNA POLYMERASE I PROMOTER ESCAPE	31	−1.86	< 0.001	0.383
REACTOME	NUCLEOSOME ASSEMBLY	18	−1.85	< 0.001	0.323
GOBP	CENP-A CONTAINING NUCLEOSOME ASSEMBLY	16	−1.81	0.006	0.449
GOBP	PYRIMIDINE NUCLEOTIDE METABOLIC PROCESS	18	−1.80	0.002	0.406
GOBP	TRANSCRIPTION ELONGATION FROM RNA POLYMERASE I PROMOTER	23	−1.78	< 0.001	0.467
GOBP	CENP-A CONTAINING CHROMATIN ORGANIZATION	16	−1.77	< 0.001	0.461
GOBP	CHROMATIN REMODELING AT CENTROMERE	18	−1.77	0.002	0.438
GOBP	DEOXYRIBONUCLEOTIDE METABOLIC PROCESS	18	−1.75	0.012	0.510
REACTOME	BASE EXCISION REPAIR	38	−1.74	0.002	0.498
GOBP	HISTONE EXCHANGE	20	−1.74	0.004	0.460
REACTOME	FORMATION OF THE EARLY ELONGATION COMPLEX	32	−1.74	0.008	0.452
GOBP	TRANSCRIPTION INITIATION FROM RNA POLYMERASE I PROMOTER	25	−1.73	0.010	0.436
GOBP	CENTROMERE COMPLEX ASSEMBLY	22	−1.73	0.006	0.425
GOBP	7-METHYLGUANOSINE RNA CAPPING	29	−1.72	0.008	0.432
REACTOME	TRANSCRIPTIONAL REGULATION BY SMALL RNAS	46	−1.71	0.008	0.472
REACTOME	RNA POL II CTD PHOSPHORYLATION AND INTERACTION WITH CE DURING HIV INFECTION	26	−1.70	0.004	0.512
GOBP	RNA CAPPING	30	−1.70	0.002	0.491
REACTOME	MRNA CAPPING	28	−1.70	0.010	0.479
REACTOME	RNA POL II CTD PHOSPHORYLATION AND INTERACTION WITH CE	26	−1.69	0.006	0.486
